# Grand Challenges in Rhinology

**DOI:** 10.3389/falgy.2020.584518

**Published:** 2020-11-23

**Authors:** Glenis K. Scadding

**Affiliations:** University College London Hospitals National Health Service Foundation Trust, London, United Kingdom

**Keywords:** allergic rhinitis, non-allergic rhinitis, chronic rhinosinusitis (CRS), chronic rhinosinusitis with nasal polyposis, chronic rhinosinusitis without nasal polyps (CRSsNP), nasal hyper-reactivity, United airways disease, olfaction

## Importance of Nasal Problems

The nose is our respiratory interface with the exterior world and, as such, bears the brunt of its challenges. The nasal and sinus epithelium, with its mucociliary clearance and production of nitric oxide gas, constitutes the first line of physical, chemical, and immunological defense of the airways, protecting us from the environment. Rhinitis and rhinosinusitis are universally experienced conditions, thanks to viral colds. This has led to trivialization of nasal symptoms and disease, even when persistent; however the realization that nasal disease is very costly to society, impairs sleep, quality of life, and work/school ability and that it is associated with a variety of co- morbidities involving the throat, lungs, sinuses, ears, and general well- being is gaining traction and leading to good quality research into etiology, prevention, and management. Many challenges remain into the etiology, pathophysiology, natural history, and therapy of upper respiratory tract disorders.

## Allergic Rhinitis (AR)

AR is a highly prevalent global health problem, found in around a quarter of European populations (1). It has a significant negative impact on quality of life (QoL) of sufferers (1–6), reducing all areas of school and work performance (7, 8), impairing mood, cognitive, and driving ability (9, 10) probably *via* its effects on reduction of sleep quality (11).

Socioeconomic effects of AR are huge (4, 5). A recent survey in Sweden found an annual cost of €1.3 billion, mainly due to presenteeism (12). AR has a negative impact on work productivity greater than that of heart disease and diabetes combined (13), hence the European Union prioritization of its control (14). In addition AR predisposes to new asthma and has deleterious effects upon existing asthma (15).

Treatment of AR is no longer simple as the disease has become more severe and complex in recent decades with many sufferers being polysensitized (8, 16) having moderate/severe disease (1, 17, 18) and persistent symptoms (19). Mixed forms of rhinitis have appeared, with a combination of allergic and non-allergic factors involved (20). In some countries local AR (i.e., localized nasal allergic response in the absence of systemic atopy, LAR) has been demonstrated (21–23). AR is among those conditions included in severe chronic upper airway disease (SCUAD, uncontrolled disease despite guideline-directed care) (24, 25) since, despite a plethora of available treatments and suitable guidelines (26, 27) AR often remains uncontrolled (28). The reasons for this are several, with patient, physician, disease, and treatment factors possibly involved. For such AR patients allergen immunotherapy may be an important treatment option (29). This more personalized treatment involves administering allergen extract, subcutaneously or sublingually, to desensitize patients, reducing both symptoms and medication requirements (30). Both subcutaneous immunotherapy (SCIT) and sublingual immunotherapy (SLIT) have been shown to be effective in patients with seasonal and perennial AR in the real world as well as in clinical trials (31) and are recommended in guidelines (29, 30). Data suggests that immunotherapy for AR in children can prevent new sensitizations and disease progression to asthma (31, 32). Questions remain about the best formulations of allergens and as yet there is still a search for biomarkers associated with, and predictive of, a successful response (33, 34).

The pathogenesis of AR is a classic example of allergic disease initiated by immunological sensitization to an allergen, probably initially in the nasal mucosa (35). The factors leading to this are both genetic and environmental, with the marked increase in AR prevalence over the last five decades probably relating to changes in the environment. Impairment of mucociliary clearance and increased epithelial permeability secondary to chemicals, pollutants, and biological factors probably allow access of molecules to the immune system, instead of their being swept away, swallowed and rendered harmless in the gut. Recent interest has also centered on the microbiome, following on from the hygiene hypothesis (36, 37). In the epidemiological ISAAC study (38) AR—associated factors, mostly shared with those of asthma and atopic dermatitis, were antibiotics, paracetamol, air pollution, farm animals in affluent countries, cats, and/or dogs, maternal, and paternal smoking, fast foods, vigorous physical activity in adolescents, frequent TV viewing, but not obesity. However, air pollution and passive smoking probably increase AR severity rather than contributing to its development (39) and few of the other associations have proven to be genuine risk factors (40).

Prevention of AR has proved difficult. There is some evidence for its reduction by exposure to farm animals in early life, and possibly to dogs, but results differ between developed and developing countries (41–43). Probiotics and /or prebiotics failed to reduce AR when given pre and post-natally (44, 45).

In recent years large scale data from mobile apps is revealing interesting facts, such as the separate nature of rhinitis with and without eye inflammation (rhinoconjunctivitis) and of rhinitis with multiple atopic co- morbidities, with involvement of different gene complexes in each of these (46).

Other areas of interest about which there is a need for more good data are non-allergic rhinitis and nasal hyperreactivity, both of which cause significant morbidity and quality of life impairment, whilst proving hard to treat effectively in many sufferers (27, 47).

Rhinosinusitis, in which inflammation extends to the sinus linings, is also a highly prevalent, quality of life reducing, condition which can be acute or chronic. The latter, abbreviated as CRS, is a poly-phenotypic entity which has been extensively reviewed recently (48) with mention of research needs in this area. This includes management of CRS with and without nasal polyps, better, large randomized, controlled trials; real-life studies combining surgery plus medical treatment, the place of biologicals, management of uncontrolled disease, impact of the extent of surgery, identification of endotypes with particular management implications.

## Links With Asthma

The concept of one airway one disease has become largely accepted—with the demonstration that most asthma patients have some form of upper airway disorder that can precede, affect and aid in classification of their lung disease (15). Rhinitis is associated with more asthma-related GP visits and hospital referrals, with associated higher costs, probably because it impairs asthma control to a similar degree as does smoking (49–51). Effective AR and CRS treatment, both medical and surgical, can improve asthma control (52–55). Prevention of allergic asthma development by allergen immunotherapy to AR sufferers has been shown with both SCIT and SLIT (31, 32), but not yet absolutely proven and the endotype for success with such therapy remains obscure.

The current requirements for asthma drug development mean that upper respiratory tract results in an asthma trial are rarely included in their major assessments- only the lower airway is monitored, whilst conversely separate upper respiratory tract studies done for nasal polyposis largely ignore asthma (56, 57). In real life many subjects have both conditions- so it is to be hoped that real life monitoring of new drugs, particularly very expensive monoclonal antibodies, will cover the whole respiratory tract.

## Surgery

For many years the upper respiratory tract has been the preserve of the ENT surgeon. Research efforts in this area tended to be sparse and of poor quality. This has changed with the advent of a new generation of scientifically trained surgeons with better investigative tools, including endoscopes and CT scans (58). The ease of access to the nose means that it has proved useful, *via* secretions, scrapings, and biopsies, in enhancing the understanding of mechanisms in the allergic and immune responses. Surgery as treatment for CRS is under investigation, with further studies ongoing (59, 60).

## Olfaction

Besides being a route for air entry and defense, the nose also provides us with the origins of the sense of smell, with cilia from olfactory receptors projecting from the brain through the cribriform plate of the skull and into the roof of the nose. Smell and its counterpart, flavor, is highly important to life and to well-being, but, like rhinitis, is often disregarded. Olfactory fibers radiate widely in the brain, particularly involving the limbic system, so smell is linked to memory and is highly emotive. Its loss can have marked impact causing feelings of isolation and reduced emotions. The resulting inability to form and maintain close personal relationships can lead to depression.

In recent years there has been increased awareness of olfaction. Olfactory receptors have been cloned and structurally assessed, leading to a Nobel prize for Linda Buck and Richard Axel (61). Development of the first of several validated tests (62) has allowed investigation of olfaction in various disorders and the demonstration of marked abnormalities in some neurodegenerative diseases (63). The olfactory pathway may represent a route for pathogens to ascend into the central nervous system (CNS) in diseases such as Parkinson's and Alzheimer's (64). Identification of malignancy or of impending epilepsy or diabetic coma by sniffing dogs has been reported (65). Most recently COVID-19 has highlighted the sense of smell. Anosmia can occur suddenly in COVID−19 sufferers (66) and may be different in several ways to that experienced in other viral upper respiratory tract infections. On current data more females and young people are being afflicted by coronavirus- induced smell loss which can be the only symptom and in around 90% is transient, suggesting that inflammation and oedema in the olfactory cleft may be responsible, rather than receptor loss.

## Conclusions

Clearly the upper airway is an area of promise for high quality research which could transform patients' lives. Thus, it forms part of the overall grand challenges in Allergy (67) which will be explored in Frontiers in Allergy.

## Author Contributions

The author confirms being the sole contributor of this work and has approved it for publication.

## Conflict of Interest

The author declares that the research was conducted in the absence of any commercial or financial relationships that could be construed as a potential conflict of interest.

## References

[1] Canonica GW Bousquet J Mullol J Scadding GK Virchow JC. A survey of the burden of allergic rhinitis in Europe. Allergy. (2007) 62(Suppl. 85):17–25. 10.1111/j.1398-9995.2007.01549.x 17927674

[2] Canonica GW Mullol J Pradalier A Didier A. Patient perceptions of allergic rhinitis and quality of life: findings from a survey conducted in Europe and the United States. World Allergy Organ J. (2008) 1:138–44. 10.1097/WOX.0b013e3181865faf 23282577 PMC3650970

[3] Nathan RA. The burden of allergic rhinitis. Allergy Asthma Proc. (2007) 28:3–9. 10.2500/aap.2007.28.2934 17390749

[4] Brozek JL Bousquet J Baena-Cagnani CE Bonini S Canonica GW Casale TB. Allergic rhinitis and its impact on asthma (ARIA) guidelines: 2010 revision. J Allergy Clin Immunol. (2010) 126:466–76. 10.1016/j.jaci.2010.06.047 20816182

[5] Brozek JL Bousquet J Agache I Agarwal A Bachert C Bosnic-Anticevich S. Allergic rhinitis and its impact on asthma (ARIA) guidelines−2016 revision. J Allergy Clin Immunol. (2017) 140:950–8. 10.1016/j.jaci.2017.03.050 28602936

[6] Price D Scadding G Ryan D Bachert C Canonica GW Mullol J. The hidden burden of adult allergic rhinitis: UK healthcare resource utilisation survey. Clin Transl Allergy. (2015) 5:39. 10.1186/s13601-015-0083-6 26583068 PMC4650835

[7] Walker S Khan-Wasti S Fletcher M Cullinan P Harris J Sheikh A. Seasonal allergic rhinitis is associated with a detrimental effect on examination performance in United Kingdom teenagers: case–control study. J Allergy Clin Immunol. (2007) 120:381–7. 10.1016/j.jaci.2007.03.034 17560637

[8] Valovirta E Myrseth SE Palkonen S. The voice of the patients: allergic rhinitis is not a trivial disease. Curr Opin Allergy Clin Immunol. (2008) 8:1–9. 10.1097/ACI.0b013e3282f3f42f 18188010

[9] Braido F Baiardini I Scichilone N Musarra A Menoni S Ridolo E. Illness perception, mood and coping strategies in allergic rhinitis: are there differences among ARIA classes of severity? Rhinology. (2014) 52:66–71. 10.4193/Rhin13.040 24618631

[10] Vuurman EF Vuurman LL Lutgens I Kremer B. Allergic rhinitis is a risk factor for traffic safety. Allergy. (2014) 69:906–12. 10.1111/all.12418 24815889

[11] Ferguson BJ. Influences of allergic rhinitis on sleep. Otolaryngol Head Neck Surg. (2004) 130:617–29. 10.1016/j.otohns.2004.02.001 15138430

[12] Cardell LO Olsson P Andersson M Welin KO Svensson J Tennvall GR. TOTALL: high cost of allergic rhinitis—a national Swedish population-based questionnaire study. NPJ Prim Care Respir Med. (2016) 26:15082. 10.1038/npjpcrm.2015.82 26845513 PMC4741287

[13] Lamb CE Ratner PH Johnson CE Ambegaonkar AJ Joshi AV Day D. Economic impact of workplace productivity losses due to allergic rhinitis compared with select medical conditions in the United States from an employer perspective. Curr Med Res Opin. (2006) 22:1203–10. 10.1185/030079906X112552 16846553

[14] Bousquet J Malva J Nogues M Manas LR Vellas B Farrell J. Operational definition of active and healthy aging (AHA): the European innovation partnership (EIP) on AHA reference site questionnaire: Montpellier October 20–21, Lisbon July 2, 2015. J Am Med Dir Assoc. (2014) 16:1020–6. 10.1016/j.jamda.2015.09.004 26498697

[15] Scadding G Walker S. Poor asthma control?–then look up the nose. The importance of co-morbid rhinitis in patients with asthma. Prim Care Respir J. (2012) 21:222–8. 10.4104/pcrj.2012.00035 22643359 PMC6547933

[16] Maurer M Zuberbier T. Undertreatment of rhinitis symptoms in Europe: findings from a cross-sectional questionnaire survey. Allergy. (2007) 62:1057–63. 10.1111/j.1398-9995.2007.01367.x 17581263

[17] Bousquet PJ Demoly P Devillier P Mesbah K Bousquet J. Impact of allergic rhinitis symptoms on quality of life in primary care. Int Arch Allergy Immunol. (2013) 160:393–400. 10.1159/000342991 23183377

[18] Demoly P Bousquet PJ Mesbah K Bousquet J Devillier P. Visual analogue scale in patients treated for allergic rhinitis: an observational prospective study in primary care: asthma and rhinitis. Clin Exp Allergy. (2013) 43:881–8. 10.1111/cea.12121 23889242

[19] Bousquet J Annesi-Maesano I Carat F Leger D Rugina M Pribil C. Characteristics of intermittent and persistent allergic rhinitis: DREAMS study group. Clin Exp Allergy. (2005) 35:728–32. 10.1111/j.1365-2222.2005.02274.x 15969662

[20] Bernstein JA. Allergic and mixed rhinitis: epidemiology and natural history. Allergy Asthma Proc. (2010) 31:365–9. 10.2500/aap.2010.31.3380 20929601

[21] Rondon C Campo P Togias A Fokkens WJ Durham SR Powe DG. Local allergic rhinitis: concept, pathophysiology, and management. J Allergy Clin Immunol. (2012) 129:1460–7. 10.1016/j.jaci.2012.02.032 22516477

[22] Rondon C Campo P Zambonino MA Blanca-Lopez N Torres MJ Melendez L. Follow-up study in local allergic rhinitis shows a consistent entity not evolving to systemic allergic rhinitis. J Allergy Clin Immunol. (2014) 133:1026–31. 10.1016/j.jaci.2013.10.034 24332860

[23] Rondon C Campo P Eguiluz-Gracia I Plaza C Bogas G Galindo P. Local allergic rhinitis is an independent rhinitis phenotype: the results of a 10-year follow-up study. Allergy. (2018) 73:470–8. 10.1111/all.13272 28833265

[24] Bousquet J Bachert C Canonica GW Casale TB Cruz AA Lockey RJ. Unmet needs in severe chronic upper airway disease (SCUAD). J. Allergy Clin Immunol. (2009) 124:428–33. 10.1016/j.jaci.2009.06.027 19660803

[25] Bousquet PJ Bachert C Canonica GW Casale TB Mullol J Klossek JM. Uncontrolled allergic rhinitis during treatment and its impact on quality of life: a cluster randomized trial. J Allergy Clin Immunol. (2010) 126:666.e1–5–8.e1–5. 10.1016/j.jaci.2010.06.034 20816198

[26] Bousquet J Khaltaev N Cruz AA Denburg J Fokkens WJ Togias A. Allergic rhinitis and its impact on asthma (ARIA) 2008 update (in collaboration with the World Health Organization, GA(2)LEN and AllerGen) *Allergy*. (2008) 63(Suppl. 86):8–160. 10.1111/j.1398-9995.2007.01620.x 18331513

[27] Scadding GK Kariyawasam HH Scadding G Mirakian R Buckley RJ Dixon T. BSACI guidelines for the treatment of allergic and non allergic rhinitis. Clin Exp Allergy. (2017) 47:856–89. 10.1111/cea.12953 30239057

[28] Hellings PW Fokkens WJ Akdis C Bachert C Cingi C Dietz de Loos D. Uncontrolled allergic rhinitis and chronic rhinosinusitis: where do we stand today? Allergy. (2013) 68:1–7. 10.1111/all.12040 23025484

[29] Roberts G Pfaar O Akdis CA Ansotegui IJ Durham SR Gerth van Wijk R. EAACI guidelines on allergen immunotherapy: allergic rhinoconjunctivitis. Allergy. (2018) 73:765–98. 10.1111/all.13317 28940458

[30] Durham SR Penagos M. Sublingual or subcutaneous immunotherapy for allergic rhinitis? J Allergy Clin Immunol. (2016) 137:339.e10–49.e10. 10.1016/j.jaci.2015.12.1298 26853126

[31] Zielen S Devillier P Heinrich J Richter H Wahn U. Sublingual immunotherapy provides long-term relief in allergic rhinitis and reduces the risk of asthma: a retrospective, real-world database analysis. Allergy. (2018) 73:165–77. 10.1111/all.13213 28561266 PMC5763412

[32] Jacobsen L Niggemann B Dreborg S Ferdousi HA Halken S Høst A. The PAT Investigator Group. Specific immunotherapy has long-term preventive effect of seasonal and perennial asthma: 10-year follow-up on the PAT study. Allergy. (2007) 62:943–8. 10.1111/j.1398-9995.2007.01451.x 17620073

[33] Bonertz A Mahler V Vieths S. Manufacturing and quality assessment of allergenic extracts for immunotherapy: state of the art. Curr Opin Allergy Clin Immunol. (2019) 19:640–5. 10.1097/ACI.0000000000000579 31449134

[34] Shamji MH Kappen J Abubakar-Waziri H Zhang J Steveling E Watchman S. Nasal allergen-neutralizing IgG4 antibodies block IgE-mediated responses: NOVEL biomarker of subcutaneous grass pollen immunotherapy. J Allergy Clin Immunol. (2019) 143:1067–76. 10.1016/j.jaci.2018.09.039 30445057

[35] Greiner AN Hellings PW Rotiroti G Scadding GK. Allergic rhinitis. Lancet. (2011) 378:2112–22 10.1016/S0140-6736(11)60130-X 21783242

[36] Strachan DP. Hay fever, hygiene and household size. BMJ. (1989) 299:1259–60. 10.1136/bmj.299.6710.1259 2513902 PMC1838109

[37] Wold AE. The hygiene hypothesis revised: is the rising frequency of allergy due to changes in the intestinal flora? Allergy. (1998) 53(Suppl. 46):20–5. 10.1111/j.1398-9995.1998.tb04953.x 9825991

[38] Asher MI Stewart AW Mallol J Montefort S Lai CKW Aït-Khaled N. Which population level environmental factors are associated with asthma, rhinoconjunctivitis and eczema? Review of the ecological analyses of ISAAC Phase One. Respir Res. (2010) 11:8–16. 10.1186/1465-9921-11-8 20092649 PMC2831000

[39] Burte E Leynaert B Marcon A Bousquet J Benmerad M Bono R. Long-term air pollution exposure is associated with increased severity of rhinitis in 2 European cohorts. J Allergy Clin Immunol. (2020) 145:834–42. 10.1016/j.jaci.2019.11.040 31983528

[40] Wise SK Lin SY Toskala E. International consensus statement on allergy and rhinology: allergic rhinitis. Int Forum Allergy Rhinol. (2018) 8:108–352. 10.1002/alr.22070 29438600

[41] Eller E Roll S Chen C-M Herbarth O Wichmann H-E Von Berg A. Meta-analysis of determinants for pet ownership in 12 European birth cohorts on asthma and allergies: a GA2LEN initiative. Allergy. (2008) 63:1491–8. 10.1111/j.1398-9995.2008.01790.x 18721248

[42] Deckers J Lambrecht BN Hammad H. How a farming environment protects from atopy. Curr Opin Immunol. (2019) 60:163–9. 10.1016/j.coi.2019.08.001 31499321 PMC7610909

[43] Levin ME Botha M Basera W Facey-Thomas HE Gaunt B Gray CL. Environmental factors associated with allergy in urban and rural children from the South African Food Allergy (SAFFA) cohort. J Allergy Clin Immunol. (2020) 145:415–26. 10.1016/j.jaci.2019.07.048 31606483

[44] Fiocchi A Pawankar R Cuello-Garcia C Ahn K Al-Hammadi S Agarwal A. World Allergy Organization-McMaster University Guidelines for Allergic Disease Prevention (GLAD-P): probiotics. World Allergy Organ J. (2015) 8:4. 10.1186/s40413-015-0055-2 25628773 PMC4307749

[45] Cuello-Garcia CA Fiocchi A Pawankar R Yepes-Nuñez JJ Morgano GP Zhang Y. World Allergy Organization-McMaster University Guidelines for Allergic Disease Prevention (GLAD-P): prebiotics. World Allergy Organ J. (2016) 9:10. 10.1186/s40413-016-0102-7 26962387 PMC4772464

[46] Bousquet J Devillier P Anto JM Bewick M Haahtela T Arnavielhe S. Daily allergic multimorbidity in rhinitis using mobile technology: a novel concept of the MASK study. Allergy. (2018) 73:1622–31. 10.1111/all.13448 29569295

[47] Sin B Togias A. Pathophysiology of allergic and nonallergic rhinitis. Proc Am Thorac Soc. (2011) 8:106–14. 10.1513/pats.201008-057RN 21364228

[48] Fokkens WJ Lund VJ Hopkins C Hellings PW. European position paper on rhinosinusitis and nasal polyps 2020. Rhinology. (2020)58(Suppl. S29):1–464 10.4193/Rhin20.600 32077450

[49] Crystal-Peters J Neslusan CA Smith MW Togias A. Health care costs of AR-associated conditions vary with allergy season. Ann Allergy Asthma Immunol. (2002) 89:457–62. 10.1016/S1081-1206(10)62081-9 12452202

[50] Price D Zhang Q Kocevar VS Yin DD Thomas M. Effect of a concomitant diagnosis of AR on asthma-related health care use by adults. Clin Exp Allergy. (2005) 35:282–7. 10.1111/j.1365-2222.2005.02182.x 15784104

[51] Clatworthy J Price D Ryan D Haughney J Horne R. The value of self-report assessment of adherence, rhinitis and smoking in relation to asthma control. Prim Care Respir J. (2009) 18:300–5 10.4104/pcrj.2009.00037 19562233 PMC6619365

[52] Watson WTA Becker AB Simons FER. Treatment of allergic rhinitis with intranasal corticosteroids in patients with mild asthma: effect on lower airway responsiveness. J Allergy Clin Immunol. (1993) 91:97–101 10.1016/0091-6749(93)90301-U 8423275

[53] Crystal-Peters J Neslusan C Crown WH Torres A. Treating allergic rhinitis in patients with comorbid asthma: the risk of asthma-related hospitalizations and emergency department visits. J Allergy Clin Immunol. (2002) 109:57–62. 10.1067/mai.2002.120554 11799366

[54] Ragab S Scadding GK Lund VJ Saleh H. Treatment of chronic rhinosinusitis and its effects on asthma. Eur Respir J. (2006) 28:68–74. 10.1183/09031936.06.00043305 16510462

[55] Howe R Mirakian RM Pillai P Gane S Darby YC Scadding GK. Audit of nasal lysine aspirin therapy in recalcitrant aspirin exacerbated respiratory disease. World Allergy Organ J. (2014) 7:18. 10.1186/1939-4551-7-18 25097720 PMC4114086

[56] Klaus F Rabe KF Nair P Brusselle G Maspero JF Castro M. Efficacy and safety of dupilumab in glucocorticoid-dependent severe asthma. N Engl J Med. (2018) 378:2475–85 10.1056/NEJMoa1804093 29782224

[57] Kim J Naclerio R. Therapeutic potential of dupilumab in the treatment of chronic rhinosinusitis with nasal polyps: evidence to date. Ther Clin Risk Manage. (2020) 16:31–7. 10.2147/TCRM.S210648 32158216 PMC6986240

[58] Scadding G Hellings P Alobid I Bachert C Fokkens W van Wijk GR. Diagnostic tools in rhinology EAACI position paper. Clin Transl Allergy. (2011) 1:2. 10.1186/2045-7022-1-2 22410181 PMC3294630

[59] Ragab SM Lund VJ Scadding G. Evaluation of the medical and surgical treatment of chronic rhinosinusitis: a prospective, randomised, controlled trial. Laryngoscope. (2004) 114:923–30. 10.1097/00005537-200405000-00027 15126758

[60] Hopkins C Slack R Lund V Brown P Copley L Browne J. Long-term outcomes from the English national comparative audit of surgery for nasal polyposis and chronic rhinosinusitis. Laryngoscope. (2009) 119:2459–65. 10.1002/lary.20653 19780032

[61] Buck L Axel R. A novel multigene family may encode odorant receptors: a molecular basis for odor recognition. Cell. (1991) 65:175–87. 10.1016/0092-8674(91)90418-X 1840504

[62] Doty RL Shaman P Dann M. Development of the University of Pennsylvania Smell Identification Test: a standardized microencapsulated test of olfactory function. Physiol Behav.(1984) 32:489–502. 10.1016/0031-9384(84)90269-5 6463130

[63] Doty R. L. (2012). Olfaction in Parkinson's disease and related disorders. Neurobiol. Dis. 46, 527–552. 10.1016/j.nbd.2011.10.026 22192366 PMC3429117

[64] Doty RL. The olfactory vector hypothesis of neurodegenerative disease: is it viable? Ann Neurol. (2008) 63:7–15. 10.1002/ana.21327 18232016

[65] Willis CM Church SM Guest CM Cook WA McCarthy N Bransbury AJ. Olfactory detection of human bladder cancer by dogs: proof of principle study [with commentary by T Cole]. BMJ. (2004) 329:712–5. 10.1136/bmj.329.7468.712 15388612 PMC518893

[66] Lechien JR Chiesa-Estomba CM Place S Laethem YV Cabaraux P Mat Q. Clinical and epidemiological characteristics of 1420 European patients with mild-to-moderate coronavirus disease 2019. J Int Med. (2020) 288:35–344. 10.1111/joim.13089 32352202 PMC7267446

[67] Papadopoulos NG. Current grand challenges in allergy. Front Allergy. (2020) 1:547654. 10.3389/falgy.2020.547654 PMC8974771 35386930

